# Comparison of Contact Patterns Relevant for Transmission of Respiratory Pathogens in Thailand and the Netherlands Using Respondent-Driven Sampling

**DOI:** 10.1371/journal.pone.0113711

**Published:** 2014-11-25

**Authors:** Mart L. Stein, Jim E. van Steenbergen, Vincent Buskens, Peter G. M. van der Heijden, Charnchudhi Chanyasanha, Mathuros Tipayamongkholgul, Anna E. Thorson, Linus Bengtsson, Xin Lu, Mirjam E. E. Kretzschmar

**Affiliations:** 1 Julius Center for Health Sciences and Primary Care, University Medical Center Utrecht, Utrecht, The Netherlands; 2 Centre for Infectious Disease Control, National Institute for Public Health and the Environment, Bilthoven, The Netherlands; 3 Centre for Infectious Diseases, Leiden University Medical Centre, Leiden, The Netherlands; 4 Faculty of Social and Behavioural Sciences, University Utrecht, Utrecht, The Netherlands; 5 Southampton Statistical Sciences Research Institute, University of Southampton, Southampton, United Kingdom; 6 Department of Microbiology, Faculty of Public Health, Mahidol University, Bangkok, Thailand; 7 Department of Epidemiology, Faculty of Public Health, Mahidol University, Bangkok, Thailand; 8 Department of Public Health Sciences, Karolinska Institutet, Stockholm, Sweden; 9 College of Information System and Management, National University of Defense Technology, Changsha, China; 10 Flowminder Foundation, Stockholm, Sweden; University of Melbourne, Australia

## Abstract

Understanding infection dynamics of respiratory diseases requires the identification and quantification of behavioural, social and environmental factors that permit the transmission of these infections between humans. Little empirical information is available about contact patterns within real-world social networks, let alone on differences in these contact networks between populations that differ considerably on a socio-cultural level. Here we compared contact network data that were collected in the Netherlands and Thailand using a similar online respondent-driven method. By asking participants to recruit contact persons we studied network links relevant for the transmission of respiratory infections. We studied correlations between recruiter and recruited contacts to investigate mixing patterns in the observed social network components. In both countries, mixing patterns were assortative by demographic variables and random by total numbers of contacts. However, in Thailand participants reported overall more contacts which resulted in higher effective contact rates. Our findings provide new insights on numbers of contacts and mixing patterns in two different populations. These data could be used to improve parameterisation of mathematical models used to design control strategies. Although the spread of infections through populations depends on more factors, found similarities suggest that spread may be similar in the Netherlands and Thailand.

## Introduction

The spread of the majority of infectious diseases, including those with pandemic potential, depends on the underlying social contact network of the host population [1], [2]. In many countries pandemic preparedness and control measures during large-scale outbreaks are analysed using mathematical models to provide support for public health policy decisions. These models often assume proportionate mixing in a population that is stratified by contact rate [3]. In many models with heterogeneity in contact rates the proportionate mixing assumption is used for convenience and because information about mixing patterns by contact rate is usually not available. In view of results that show that mixing patterns between susceptible individuals greatly influence infectious disease dynamics [4]–[6], it is pertinent to collect empirical information about mixing patterns that determine the transmission of infectious diseases.

Most studies that investigated contact patterns relevant for the spread of respiratory infections that are spread by close-contact used egocentric data, i.e. responses of participants who were sampled independently of one another [7]. Such studies provide no information about the contact network structure beyond the contact persons reported by participants. Electronic proximity devices can provide more insight in social contact networks, but their use is limited to specific settings [8], [9]. Although complete analysis of social contact networks is practically impossible for an entire population, partial insight in degree distributions and contact mixing patterns within these networks can increase our understanding of disease dynamics [10], [11]. For example, when individuals primarily have contact with individuals of similar age, infections are likely to spread faster within those subgroups than among them. Furthermore, investigating contact networks can highlight the existence of so-called hubs, i.e. individuals with a relatively high degree [12], who are more likely to become infected and to infect others, and can act as a bridge between communities. Such information can be used to determine optimal control strategies.

Respondent-driven sampling (RDS) is a chain-referral method that was introduced to estimate disease prevalence in hard-to-reach populations, such as drug-users [13], [14]. RDS begins with the selection of initial respondents, so-called “seeds”, who are asked to recruit contact persons from their social environment; these contact persons are asked to do the same. Unlike snowball sampling, RDS provides the option to track who recruited whom by means of personal codes. Previously, we proposed online RDS (webRDS) as a method for sampling contacts of contacts and beyond of individuals to study social contact network structures [15]. To investigate the determinants of using this sampling method for eliciting contact information, we conducted webRDS surveys in both the Netherlands and Thailand focusing on contact patterns relevant for the transmission of respiratory pathogens. Note that our objective was not to use RDS for estimating population proportions from our samples.

In this paper, we compare social contact data that were collected with a similar methodology in the Netherlands and Thailand. Earlier, a large population-based study showed striking similarities in mixing patterns across eight European countries [16], and also comparable egocentric data on mixing patterns were collected in countries in Asia [17], [18]. However, to our knowledge, no studies performed a direct comparison between contact network data collected in a European country and a country in Asia. Our first aim was to study whether populations with different cultural, demographic and social backgrounds also differ in social mixing patterns. In particular, we aimed to investigate differences and similarities in numbers of contact persons, mixing patterns (i.e. who has contact with whom?) and effective contact rates between both countries. The latter help quantifying the transmission potential of certain directly transmitted infectious diseases through a population. Secondly, our aim was to investigate whether there are differences between the two study populations with respect to factors that drive peer-recruitment via the internet.

## Methods

We conducted webRDS surveys to collect contact network patterns in Thailand and the Netherlands between November 2012 and May 2013 (see Figure S1). Recruitment methods and questionnaires were virtually identical in both countries (apart from different survey languages and minor tailoring to local situations that is elaborated below), in order to carry out a country comparison. Seeds were invited from convenience samples of students from two universities located in Utrecht and Amsterdam (the Netherlands) and two Bangkok Universities (Thailand) (Table 1). The majority of the students in both countries leave the parental home and live in dormitories [19], [20]. In the Netherlands it is custom for a student to have an own bedroom but to share all facilities, whilst students in Thailand often share the same room or even the same bed. Seeds in both countries were first informed about the survey, either in physical group meetings or by an information email. Seeds were then invited by email containing a unique hyperlink to the questionnaire or they could register themselves on the survey website using their email address. The questionnaire and invitation emails were provided in local languages and additionally in English.

**Table 1:** Characteristics study populations and recruitment.

		Netherlands	Thailand
**Participation**	Total participants	358	257
	Complete responses	89.9% (322)	85.6% (220)
	Invited seeds	189	191
	Seeds who filled in questionnaire	48.7% (92)	46.6% (89)
	Seeds who successfully recruited^a^ one or more recruitees	32.8% (62)	20.4% (39)
	Pairs of recruiter-recruitee (both completed survey)	233	140
	Maximum number of waves	5	6
	Trees with two or more waves	46.8% (29)	38.5% (15)
**Successfully recruited** a **by**	Facebook	10.4% (24)	83.6% (117)
	Indirect email invitation	52.6% (121)	5.0% (7)
	Direct email invitation	37.0% (85)	11.4% (16)
**Age**	Overall mean (range)	33.2 (16–75)	26.7 (14–52)
	Mean age seeds^b^ (range)	27.7 (25–53)	26.2 (14–48)
**Sex**	Female	62.1% (221)	61.4% (156)
	Male	37.9% (135)	38.6% (98)
**Education**	Higher education	89.9% (320)	83.9% (213)
	Vocational Education/Higher diploma	4.2% (15)	0.8% (2)
	High School/Other education	5.9% (21)	15.3% (39)
**Symptoms**	Two or more symptoms	39.6% (128)	29.9% (66)
	ILI symptoms^c^	1.9% (6)	3.2% (7)
	Common cold-like symptoms^c^	7.4% (24)	5.9% (13)

^a^Successfully recruited means that the contact person completed the questionnaire.

^b^Seeds who successfully^a^ recruited one or more contact persons.

^c^ILI symptoms are a combination of the self-reported symptoms: fever, headache and muscle pain; common cold-like symptoms are a combination of the symptoms: runny nose, sore throat and cough.

### Contact definition and questionnaire

The questionnaire was kept short to limit the burden for participants; in total it consisted of eleven questions. A ‘contact person’ was defined as a person sitting or standing within arm's length of the participant (denoted as ‘YourSpace’, see Figure S2) for 30 seconds or longer. This definition made it fairly simple to recall contact persons, could be easily explained in an online questionnaire, and was not restricted to only face-to-face interactions. Participants were asked to record numbers of contact persons at different settings during one day (namely ‘yesterday’). We asked participants to record the number of contact persons while travelling and at different locations. Modes of transport and locations were prespecified (i.e. a subset of transport and location types of interest, see Table S1) in the questionnaire and tailored to each country. Participants were asked in a separate section to record the number of contact persons within arm's length while eating, as it is custom in Thai culture to share several small dishes and drinks with friends and/or family in restaurants, which facilitates the potential transmission of infections. For numbers of contact persons at different locations and while eating, the participant was requested to indicate whether the contact person was younger, the same age or older than the participant. We also asked participants for their age, sex, educational level, postal code, and the number and ages (specified in age groups) of persons living in their household during the past seven days. Furthermore, we included a question to record any symptoms (provided in a list, see Table S2) that participants and/or household members experienced in the past seven days. A combination of the symptoms fever, headache and muscle pain was indicated as influenza-like-illness (ILI) symptoms, and a combination of the symptoms runny nose, sore throat and cough as common cold-like symptoms.

### Recruitment and online system

At the end of the questionnaire, we asked participants to recruit four new participants (further referred to as ‘recruitees’) with whom they had contact according to the above definition in the past 7 days (see also Figure S2). We provided three options for recruitment, namely through Facebook (i.e. send Facebook friends a private message), indirect email (i.e. provide your email address and receive four invitation emails that can be forwarded to recruitees) and direct email (i.e. provide email addresses of your recruitees and the system sends out invitation emails automatically). All invitations contained information on the survey and a personal link and code. All emails and the first page of the questionnaire contained a link to unsubscribe for the survey.

After inviting recruitees, participants were referred to the project website [21] where participants could view a graphical representation of the network components found in the study as a non-material incentive for their participation. These network graphs were anonymous but showed the personal codes provided in the invitations so that participants could identify their own location in the network. Full details of the online RDS survey system can be found in M.L. Stein et al. [15].

### Ethical statement

All information about the survey was available on all web pages and could be accessed at any time. All pages contained a log-out button that referred users to a search engine. The system converted IP addresses to a unique anonymous code using a one-way encryption algorithm and original addresses were deleted. Login passwords were only valid for a single participation and could not be used on two computers at the same time. All communication between the users and the survey website was encrypted. We obtained informed consent via the first webpage, on which inter alia study purposes and benefits, and statements on privacy and confidentiality were displayed. Users were able to accept the informed consent form by clicking the ‘Start the Survey’ button and to continue to the questionnaire, or to deny by clicking the button “No Thanks” whereby users were automatically logged out of the survey. There was no age limit for inclusion included in the study protocols. Due to the setup of the study a selection of participants based on age or a separate consent form for parents/caretakers of minors was not possible. The study received approval from the Medical Ethical Committees from both the University Medical Center Utrecht, the Netherlands (reference: 12-247/C) and the Faculty of Public Health Mahidol University, Thailand (reference: MUPH 2012-187).

### Statistical analysis

Using terminology from social network analysis, we defined a participant's degree as the total number of contact persons reported by the participant during one full recording day (i.e. the sum of the numbers of contact persons *at different locations* and *while travelling*). We removed two Thai participants who reported a degree of more than 2200, as these extreme degree values are highly unlikely according to our contact definition and their individual answers made clear that they misinterpreted the definition. A negative binomial distribution N (*μ*, *k*) was fitted to the observed frequency distribution of degrees using maximum likelihood methods, where *μ* denotes the mean and *k* the dispersion parameter; parametric bootstrapping was performed to estimate confidence intervals. We used a Q-Q plot to graphically compare the degree distributions of the two country samples and used the Anderson-Darling *k*-sample test to test the hypothesis that these two samples come from one common population.

To investigate mixing patterns within our two samples with respect to the measured variables, we calculated correlation coefficients between pairs of randomly chosen individuals that were one, two, three and four or more link steps away from each other in the same network chain, by using the shortest paths between any two persons in the same network chain [11]. Thus, we calculated correlations between recruiters and their recruitees in consecutive waves. Here we assumed that a recruitment link between two participants can be interpreted as a contact in the sense of our contact definition. We used Pearson's *r* for integer variables (age, degree, and number of contact persons while eating), phi coefficient (*r* ***_φ_***) for binary variables (sex and two or more symptoms reported) and Spearman rank-order (*r*_rank_) for ordinal variables (education).

Logistic regression analysis was used to investigate which measured characteristics are important for online peer-recruitment. We defined the binary outcome “intention to recruit” as a respondent that requested invitations for recruitees on the last survey page (versus a respondent who did not request invitations). We used this binary outcome as the RDS system only registered whether or not a participant clicked the button to request for four invitations, and not how many of the four invitations were actually sent out to recruitees. We repeated the logistic regression analysis for the sampled data without seeds (data without wave 0), to investigate the influence of seeds on the outcome. See Text S1 for a more detailed description. In RDS methodology, samples are weighted for individual degrees as persons with a high degree theoretically have a higher chance to get invited than persons with a low degree [22]. For illustration purposes, we used the output of the logistic regression model to estimate for individual subjects in each country the probability of the intention to recruit as a function of degree; adjusted for age, sex, education and household size. Confidence intervals (95%) were obtained using standard errors.

In addition, we assessed for each country sample the validity of the first-order Markov assumption [23], i.e. that correlations found between recruiter and recruitee are only dependent on the direct recruiter. This was done by calculating the correlations for age and sex between seeds (wave 0) and their recruitees in consecutive waves (maximum up to 3 waves, due to limited number of participants in waves ≥4). For the numeric variable “age” we compared *r*_waves0–3_ with *r*_waves0–1_ * *r*_waves1–2_ * *r*_waves2–3_; for the categorical variable “sex” we raised the 1-step transition matrix to the third power to obtain *r*_waves0–3_.

### Effective contact rate

In a population that is heterogeneous with respect to contact rates, the basic reproduction number depends on the so-called effective contact rate *C*. Assume that a population is stratified into *n* subgroups with contact rates c_i_, i = 1, 2, 3,…,*n*. Furthermore, assume that mixing is proportionate, i.e. random mixing weighted by the contribution of a subgroup to the total number of contacts in the population. Then it can be shown that the basic reproduction number R_0_ is proportional to the effective contact rate given by
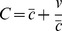
(1)where 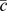 denotes the population mean of the c_i_ and *v* the variance of the c_i_ (see Anderson and May [3]; p 233).

Here we used the concept of the effective contact rate to quantify the heterogeneity in contact rates found in our sample and to assess their possible impact on transmission of infection. Based on the assumption that degree distributions observed in our sample are representative for the degree distribution in the population, we computed *C* with data on degree. In addition, we computed an effective contact rate with contact persons at different locations, contact persons while travelling, contact persons while eating and household members in order to compare and assess the contribution of each of these categories. This provides some indication for the possible effects of control measures that reduce contact rates such as school closure or cancellation of mass gathering events on transmission of infection [24]. We are aware that the assumption of representativeness of our sample of the degree distribution in the population is most likely not fulfilled, but want to demonstrate how such data can be used to investigate the impact of interventions on social network connectivity.

**Figure 1:**
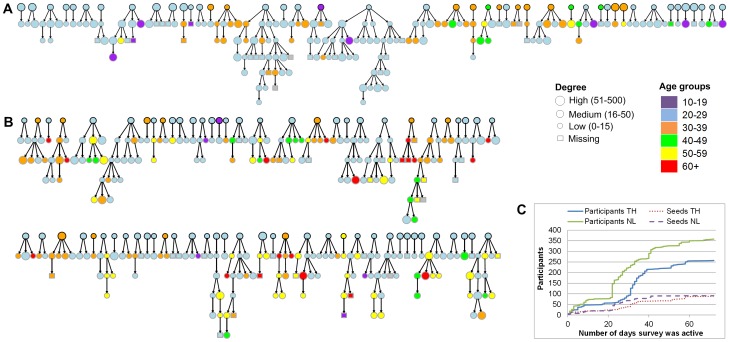
RDS networks and participation over time. The recruitment networks obtained in (**A**) Thailand and (**B**) the Netherlands. Seeds are indicated with black borders; seeds who did not recruit other participants are not displayed. The circle sizes reflect the total number of contact persons (degree) reported by each participant. The colours indicate the different age groups, illustrating the mixing by age. (**C**) Displays the cumulative number of participants and seeds over time the surveys were active in each country. The jumps in the cumulative number of participants are caused by newly invited seeds.

We also note that the applied statistical tests do not take the interdependence structure within our sampled data into account. The RDS data files are available online, doi: 10.6084/m9.figshare.1147465. R version 3.0.3 was used for statistical analysis and RGraphviz for creating Figure 1. See Text S2 for the full R source code and applied libraries.

## Results

**Figure 2:**
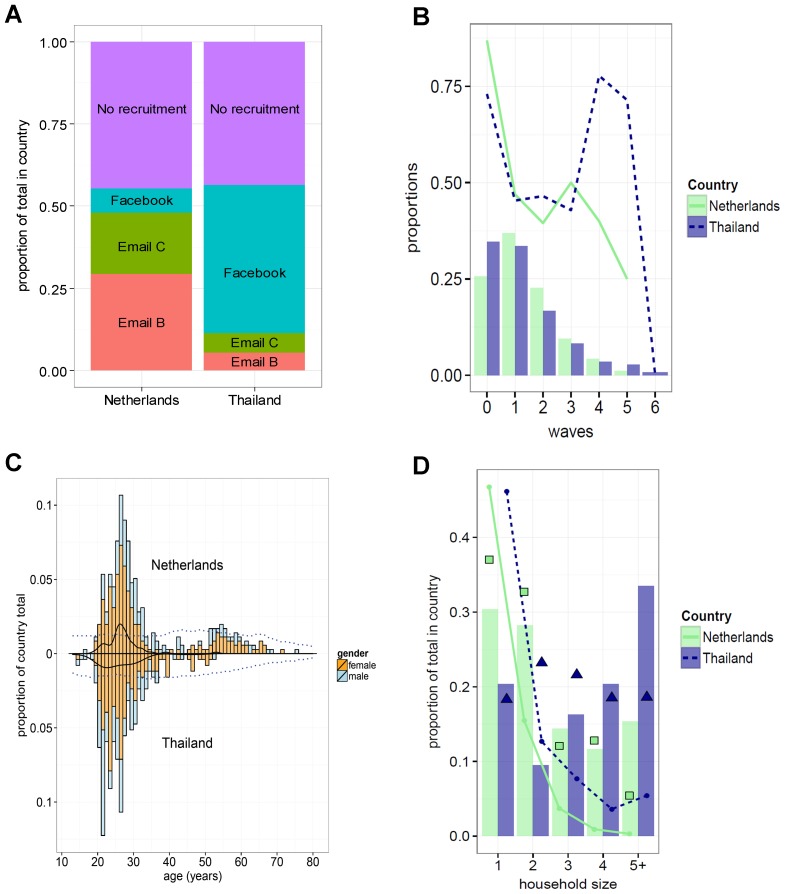
Characteristics of study participants and recruitment. (**A**) Recruitment options used by participants. (**B**) Distribution of participants over waves (in proportions of country totals). The lines indicate the proportion of participants, in each wave, who also invited recruitees (these participants requested four invitations on the last page of the survey). In both countries the proportion of seeds (wave 0) that invited recruitees was more than 70%, and on average more than half of all participants invited one or more recruitees. (**C**) The age distributions in each country for all participants, with colour indicating sex. The solid lines (black) display the age distributions for seeds only. The blue dotted lines show the estimated Dutch and Thai population estimates for 2012. (**D**) The household sizes reported by participants in each country (bars). The squares (for the Netherlands) and triangles (Thailand) show the household sizes as reported by the national census bureaus. The lines in the graph indicate the number of household members with one or more symptoms (as a proportion of the country totals), as reported by participants (30 participants in the Netherlands did not known whether household members had any symptoms, compared to 20 in Thailand).

A total of 358 individuals participated in the Netherlands, compared to 257 participants in Thailand. Although in both countries the survey's operating days, numbers of invited seeds and seeds who filled in the questionnaire were similar, seeds in the Netherlands were more successful in inviting recruitees who also completed the questionnaire (see Table 1). Nevertheless, in Thailand we reached up to six waves (in two trees) of recruitees, compared to five waves (in three trees) in the Netherlands (see Figure 1). There were 233 pairs of recruiter-recruitee in the Netherlands and 140 in Thailand. In both countries, more than half of all participants invited recruitees, namely 55.4% (198) in the Netherlands and 56.4% (145) in Thailand. Of all seeds, 87.0% (80) in the Netherlands invited one or more recruitees compared to 73.0% (65) in Thailand. The majority of the Thai participants (45.1% of 257 participants) used Facebook to invite recruitees, while in the Netherlands invitations were mainly sent by email (Figure 2).

### Characteristics of participants

The age distributions of seeds in both countries differed significantly (K-S test *p* = 0.018); with seeds in the Netherlands having a mean age of 26.7 (median: 26.0, range: 19–53) and in Thailand 26.2 (median: 24.0, range: 14–48). In the Netherlands the relatively younger participants more often recruited individuals from an older generation, which led to a wider range of participating age classes. Consequently, the entire Dutch sample had a higher overall mean age (33.2, median: 28.0) compared to Thailand (26.7, median: 25.0). The age distributions of all participants in both countries differed significantly (K-S test *p*<0.001).

The majority of participants in both countries was female and highly educated (Table 1). The Dutch sample contained 11.6% more females compared to Dutch national census data, in the Thai sample there were 10.4% more females than in the national census data from Thailand [25]. Figure 2 displays the age distributions of all participants, the age distribution of all seeds who invited one or more recruitees, and the distributions by sex of all participants in both countries. Dutch participants reported more often living in households consisting of only one or two members (by 58.6% of 326), while in Thailand household sizes of three or more persons were more often reported (by 70.1% of 221). The distribution of reported household sizes was roughly in agreement with national census data (Figure 2d). However, compared to census data, both country samples contained higher proportions of participants living in a household of five or more members, and the Thai sample contained relatively low proportions of participants living in a two-member household [25], [26].

### Self-reported symptoms

Participants in the Netherlands reported 15.7% more symptoms and 1.5% more common cold-like symptoms than Thai participants, but in Thailand participants reported 1.3% more ILI symptoms (Table 1). In Thailand, 33.5% (of 221) reported one or more household contacts with symptoms, compared to 31.6% (of 323) in the Netherlands.

### Reported numbers of contact persons

**Figure 3:**
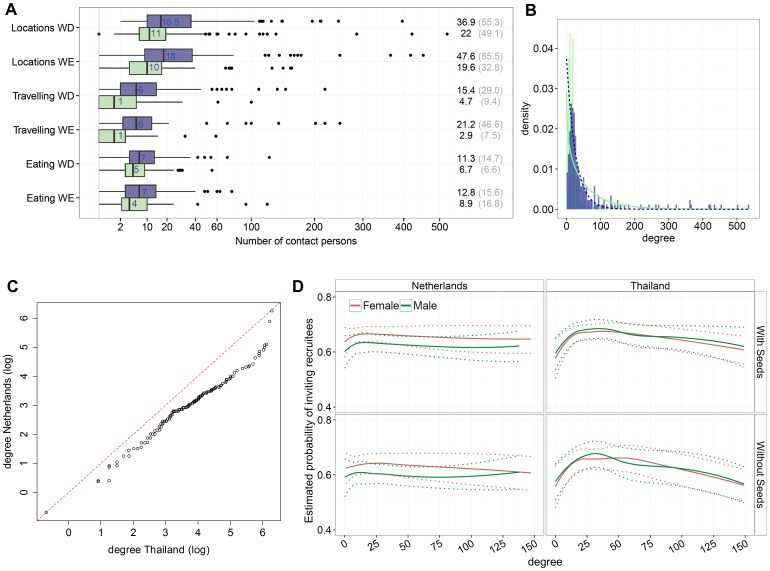
Egocentric contact data. (**A**) Box and whisker plot showing the median, quartiles and 95 percentiles of numbers of contact persons reported by participants for travelling, at different locations and while eating, during weekdays (WD, Monday-Friday) and weekend days (WE, Saturday-Sunday). Dutch sample is indicated in green and Thai sample in purple. (**B**) Distribution of the overall reported numbers of contact persons (“degree”) in each country. The green solid line (the Netherlands) and the dotted purple line (Thailand) indicate the fitted negative binomial distributions for reported degree. (**C**) Quantile-Quantile plot of the degree distributions displayed in plot 3B. (**D**) The probability estimates of inviting recruitees (“intention to recruit”) as a function of degree, specified by sex (and additionally adjusted for variables age, education and household size). Dotted lines indicated confidence intervals (95%). Values were obtained using logistic regression analysis for the full sample and the sample excluding seeds (i.e. sample without wave 0).

A total of 12812 contact persons were reported in Thailand, compared to 8336 in the Netherlands. Figure 3a shows that the medians of the distributions of numbers of contact persons at different locations, while travelling and while eating were higher in Thailand than in the Netherlands. Summing contact persons reported for each location showed no significant differences between days of the week in both samples (see Figure S3). In Thailand there were significant differences in distributions between days of the week for contact persons while travelling, e.g. between Monday and Friday (*p* = 0.036) and Saturday and Sunday (*p* = 0.014), these differences were not observed in the Netherlands (see Figure S4).

Figure 3b shows the fitted negative binomial distributions to the observed frequency distributions of degree in the Netherlands (*μ* = 25.65 [23.15–28.51]; *k* = 1.00 [0.87–1.16]) and Thailand (*μ* = 58.51 [50.30–67.24]; *k* = 0.83 [0.72–1.02]). The non-overlapping confidence intervals of the parameter estimates *μ* indicate that the sampled degree distributions were not similar, which is confirmed by visually examining the Q-Q plot (Figure 3c) and the Anderson-Darling test (*p*<0.001). Table 2 displays the means, variances and effective contact rates for the various contact categories. The higher means and variances of the observed degree distributions in Thailand indicate not only more heterogeneity, but also overall more contact persons compared to the Dutch sample. Consequently, the effective contact number computed with degree was almost two times higher in Thailand than in the Netherlands (respectively 205.5 and 111.9). Similar differences in means, variances and *C* between both countries were observed for contact persons at different locations, while travelling and while eating.

**Table 2:** Effective contact rates *C*.

		The Netherlands	Thailand
Degree (total number of contact persons)	Mean	25.6	58.5
	Variance	2212.2	8601.9
	*C*	111.9	205.5
Contact persons at different locations	Mean	21.4	40.9
	Variance	2057.6	4671.4
	*C*	117.6	155.0
Contact persons while travelling	Mean	4.2	17.6
	Variance	80.8	1346.9
	*C*	23.3	94.2
Contact persons while eating	Mean	7.3	11.8
	Variance	106.5	225.0
	*C*	21.9	30.8
Household members	Mean	3.0	4.0
	Variance	10.1	8.6
	*C*	6.4	6.1

### Drivers of online recruitment

Figure 3d shows the estimated probability of inviting recruitees as a function of degree. In Thailand, degree seemed to influence participants' intention to invite recruitees. However, when excluding Thai seeds there was no significant influence of degree, and only participant's age slightly influenced the intention to recruit. In the Netherlands, female participants requested more often the four invitations to invite their recruitees compared to male participants (*p* = 0.009, adjusted for degree, age, education and household size). Excluding seeds in the analysis showed for the Dutch sample similar results with respect to those differences between males and females. See Text S1 and Figure S5 for the extended analysis.

### Mixing patterns

**Figure 4:**
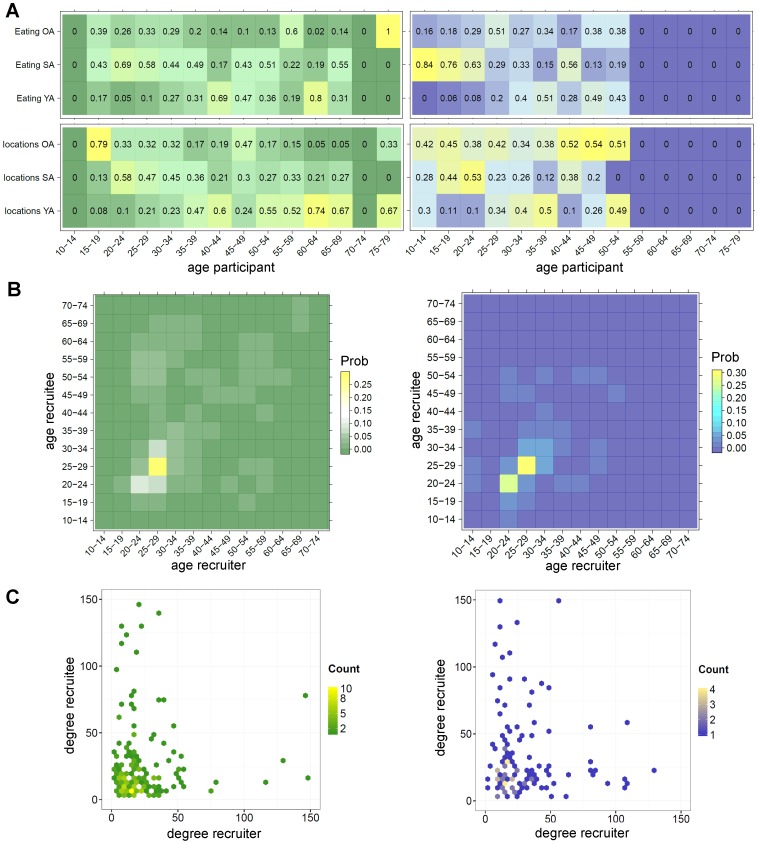
Who has contact with whom? The green coloured plots on the left are all based on data collected in the Netherlands and purple coloured plots on the right on data collected in Thailand. Plots in (**A**) display for each age group the proportion of reported contact persons that were younger, the same age or older than the participants. Displayed values are proportions that were calculated separately for the categories “contact persons while eating” and “contact persons at different locations”. The brighter coloured cells indicate higher proportions. (**B**) The contact intensity matrices for recruiter-recruitees pairs with respect to age (displayed as proportions of country totals) and (**C**) degree (displays actual reported degree; outliers above 150 are not displayed). The brighter coloured cells/points in B-C illustrate higher contact recruitment rates and the darker cells/points lower rates.

Figure 4a displays for each age group the proportions of contact persons younger than, same age or older than the participants at different locations (reported number of contact persons at different locations aggregated) and separately while eating. In both study populations, participants showed a strong tendency to invite recruitees of similar age, sex and education, although the Dutch sample showed lower correlations for mixing by education (Table 3). The assortative recruitment patterns by age, sex and education disappeared in both samples after a distance of three or more link steps between any two persons in the same network chain. However, in the Thai sample, assortative recruitment by education persisted until a distance of three link steps. By contrast, mixing by degree was observed not to be assortative (or disassortative) in both countries, but random for all link distances between two persons in the same chain. Figure 4b illustrates the mainly assortative recruitment patterns by age in each country, but also some recruitment of participants from different age classes, especially in Dutch sample. Figure 4c illustrates for both countries the random mixing by degree of recruiters-recruitees.

**Table 3:** Correlations between any two linked individuals in the same network tree.

		Number of link steps between any two individuals in the same network			
	Country^a^	1	2	3	≥4^b^
**Age (** ***r*** **)**	**NL**	0.469 [0.369–0.558]*	0.158 [0.048–0.265]£	−0.071 [−0.193–0.053]	0.021 [−0.102–0.143]
	**TH**	0.555 [0.439–0.652]*	0.350 [0.224–0.465]*	0.087 [−0.050–0.222]	0.040 [−0.039–0.118]
**Sex (** ***φ*** **)**	**NL**	0.289 [0.174–0.396]*	0.055 [−0.057–0.165]	0.103 [−0.021–0.223]	0.066 [−0.057–0.187]
	**TH**	0.205 [0.054–0.346]£	0.132 [−0.005–0.264]	−0.045 [−0.180–0.092]	0.023 [−0.054–0.100]
**Education (r** ***_rank_*** **)**	**NL**	0.133 [−0.018–0.293]£	−0.001 [−0.095–0.114]	0.037 [−0.087–0.174]	−0.050 [−0.139–0.056]
	**TH**	0.553 [0.362–0.735]*	0.336 [0.141–0.531]*	0.188 [−0.005–0.392]£	−0.071 [−0.112–−0.018]
**Degree log (** ***r*** **)**	**NL**	0.081 [−0.047–0.207]	0.049 [−0.071–0.169]	0.028 [−0.109–0.164]	−0.009 [−0.144–0.127]
	**TH**	−0.055 [−0.220–0.113]	−0.103 [−0.251–0.049]	0.119 [−0.038–0.270]	0.042 [−0.050–0.133]
**Degree - categories (** ***r_rank_*** **)**	**NL**	0.046 [−0.085–0.180]	0.128 [0.012–0.244]£	0.022 [−0.115–0.164]	−0.076 [−0.199–0.055]
	**TH**	0.049 [−0.119–0.223]	−0.027 [−0.186–0.122]	0.087 [−0.068–0.234]	0.005 [−0.080–0.095]
**Number of contact persons while eating log (** ***r*** **)**	**NL**	0.051 [−0.077–0.177]	0.020 [−0.100–0.138]	−0.011 [−0.146–0.125]	0.091 [−0.045–0.223]
	**TH**	0.167 [−0.000–0.325]	−0.007 [−0.159–0.145]	−0.020 [−0.176–0.136]	−0.002 [−0.094–0.090]
**Two or more symptoms reported (** ***φ*** **)**	**NL**	−0.018 [−0.142–0.111]	0.1314 [0.013–0.257]£	−0.019 [−0.152–0.115]	−0.010 [−0.148–0.120]
	**TH**	−0.059 [−0.208–0.115]	0.000 [−0.152–0.157]	−0.111 [−0.257–0.038]	0.001 [−0.095–0.089]

^a^NL: the Netherlands; TH: Thailand.

^b^Distances of four or more links were lumped together.

*p-value<0.001.

^£^p-value<0.05.

### First-order Markov assumption

In both the Netherlands and Thailand, the sample correlations *r*_waves0–3_ for age (respectively 0.171 and 0.320, see Table S3) were not in agreement with *r*_waves0–1_ * *r*_waves1–2_ * *r*_waves2–3_ (respectively 0.086 and 0.147). Also for sex, sample correlations (respectively 0.106 and 0.000) slightly deviated from correlations obtained from the three-step transition matrices (respectively 0.029 and 0.013). This suggests for peer-recruitment, with regard to age and sex, a higher-order process in both countries. Thus, correlations found between the recruiter-recruitee are not only dependent on the direct recruiter, but also on recruiters in previous waves. Due to a limited number of waves in most network trees, we were unable to quantify the exact order level.

## Discussion

Here we have reported on results from similar webRDS surveys conducted in the Netherlands and Thailand. Our study provides, to our knowledge, the first comparison of social contact data relevant for infections transmitted by the respiratory or close-contact route from countries in Europe and South-East Asia. Even though these two countries differ in many aspects of their cultural, social and demographic determinants, comparison of the country samples showed clear similarities in contact and mixing patterns. Mixing was assortative by age, sex and education, and random by degree. We reached a similar number of waves in both countries using the same non-material incentives. By contrast, differences between both country samples were observed for age classes reached, age of daily contact persons and levels of dispersion in overall degree distributions. Moreover, seeds in the Netherlands were more successful in inviting recruitees, and female participants in the Netherlands showed a significantly higher intention to recruit; this sex difference was not observed for the Thai sample. Thai participants primarily used Facebook, which is very popular among young Thai [27], to invite recruitees while standard email was more preferred in the Netherlands.

Despite comparable high population densities in Bangkok (5294 persons per square kilometer [26]) and the Dutch regions (3353 in Utrecht and 4767 in Amsterdam [28]), where we invited our seeds, Thai participants reported on average higher numbers of contact persons. Both study samples showed a strong heterogeneity in numbers of contact persons per individual, which was also found earlier in other populations [17], [29]. Theoretical studies have demonstrated that heterogeneity in numbers of contacts influences both the infection attack rate [30] and the basic reproduction number [31], thereby impacting on the effectiveness of control measures. It should be noted that the adopted contact definition was different from the definition generally used by contact diary surveys (i.e. two-way conversation in close proximity or a physical contact like shaking hands or kissing). This has implications for the comparability with other studies, especially when comparing location-dependent contact behaviour [32].

Our study samples showed random mixing by degree, which gives support to the proportionate mixing assumption underlying the derivation of the effective contact rate as computed in equation (1). Regarding the effectiveness of control measures our data provide some indications for assessing possible effects of interventions that reduce numbers of contacts. For instance, our data illustrate that by asking all individuals in a population to stay at home during an outbreak would reduce the effective contact rate from 111.9 to 6.4 in the Netherlands and from 205.5 to 6.1 in Thailand.

However, the question remains how well correlations between recruiters and recruitees describe the mixing patterns in social networks; this depends mainly on the randomness of peer-recruitment. In Thailand, participants invited more recruitees of similar age, sex and education, suggesting that the survey spread in a more homogeneous group compared to the Netherlands. This might be due to inequalities between urban and rural areas in Thailand, especially with respect to education, income and occupation opportunities [33]. Educated Thai are therefore more likely to connect to similarly educated peers. In the Netherlands, participants from the younger age groups more frequently invited recruitees from older age classes that led to recruitment within these older age groups. Such links between younger and older age groups were less visible in Thailand. This could either be due to social-cultural differences or to the low proportion of internet-users among Thai aged 35 and older.

Our Dutch recruiter-recruitee matrix for age showed a similar assortative pattern (including links between younger and older age groups), as the contact matrix that was collected earlier in the Netherlands during a large egocentric survey (POLYMOD, a population-based survey on social contact patterns conducted in eight European countries [34]). These similarities in contact matrices imply that recruitment links may be representative for the social network links in our Dutch study population, at least with regard to age. However, young children (aged 0–12) and network links between these children and adults are missing in our country samples. We were unable to make a similar comparison of contact matrices for Thailand, as there are no Thai contact data available yet in the published literature. A large household-structured survey conducted in Vietnam [17] (a middle-income country, also densely populated in urban areas) demonstrated similar assortative mixing patterns by age as was seen in our Thai sample.

It is important to recognize the limitations of the presented data. Our results are based on small sample sizes and are not representative of the general population. In both countries we solely invited students of which the majority was aged between 20 and 30 years. The survey remained mainly within that age group, therefore limiting the generalisability of our results to other age groups. For the analyses of mixing patterns, the numbers of contact persons that were reported by recruiters and recruitees for one single day might not be a good representation of their contact frequency during the entire week. The data do contain responses for all days of the week, such that a comparison between weekdays and weekends is possible, but these are cross-sectional data and not longitudinal information per participant. We do not have information about individual variation in numbers of contacts during the week. In addition, in both countries only a small number of recruitment trees reached more than 4 waves, which may be a consequence of using solely a non-material incentive. The use of a small material incentive to stimulate online peer-recruitment should be further investigated as was done previously for hard-to-reach populations [35], [36].

Although we chose to use an aggregated contact diary design to limit the burden for each participant and to stimulate online peer-recruitment, this prevented us from collecting other determinants relevant for infection transmission, such as contact duration and intensity. Earlier studies have shown the importance of contact duration for understanding the transmission dynamics of close-contact pathogens [37], [38]. Also, contact duration influences the probability that a contact is reported by a participant [39], [40]. Although it is possible to derive contact durations from earlier conducted diary surveys, we cannot preclude the effect of heterogeneities in motivation or recall capabilities on the reported degrees, between both country samples, and between participants in same country (e.g. differences in reporting quality between females and males [39]).

The applied survey methodology was also unable to provide information on clustering of contact persons within small subgroups of the population. Theoretically it has been demonstrated that clustering of contacts influences transmission dynamics [5], [6], [41], [42]. We did provide our participants with a separate link to indicate repeated invitations, but we received no reports of survey clustering. It remains challenging to motivate persons to undertake action in case they receive multiple invitations, as most persons instantly delete repetitive emails. In a next stage, we plan to experiment with a design in which contact persons need to be reported by name in order to measure clustering, like was done in some diary studies [39], [40]. However, this introduces new privacy issues that may prevent participants from inviting contact persons, and thereby limit the possibility to study correlations between connected individuals.

The proportions of ILI symptoms found in our samples are broadly in agreement with estimates on the incidence of seasonal influenza, at least for the Netherlands. Previously, a study estimated that the overall symptomatic infection attack rate was 2.5% for seasonal influenza in the Netherlands [43]. If all symptomatically infected persons would be symptomatic at the same time, we would expect to find similar numbers in our sample. However, infections are spread over several months (see Figure S1) and therefore the 1.9% ILI cases found in the Dutch sample seems reasonable. This proportion seems even high when comparing the period of survey administration to the total period of the influenza season, this may indicate clustering of the influenza virus. There are several factors that influence the detection of symptoms via the applied RDS methodology, e.g. immunity in host populations can be clustered. Although natural immunity is hard to measure, clustered influenza vaccination patterns have been described in literature [44], [45]. Also, participants were only asked to report any symptoms that they had in the past 7 days and it is possible that they experienced symptoms before this 7-days period or in the days after filling in the questionnaire. We are currently conducting new webRDS surveys in which we extended the 7-days period to 14 days, and added a follow-up measurement 3 weeks later to investigate whether participants developed any symptoms in the meantime.

Our findings from the comparison of data collected with RDS surveys in two countries provide new insight on contacts and mixing patterns within social networks. Information on correlations between linked individuals can be used to improve parameterisation of mathematical models used to design optimal control strategies and lend support to the often used proportionate mixing assumption. Although the spread of infections through a population depends on more factors than just contacts and mixing patterns, similarities found between both countries suggest that the spread of directly transmitted respiratory infections may be similar in the Netherlands and Thailand, and in other countries with comparable contact patterns.

## Supporting Information

Figure S1 **Period of survey administration and influenza activity in each country.** (**A**) displays the influenza activity in Thailand in numbers of specimens that were found positive for influenza (as provided by FluNet [46]). (**B**) Number of participants in Thailand collected between December 2012 and March 2013. (**C**) Number of participants in the Netherlands collected between end of November 2012 and beginning of May 2013. Most participants filled in the questionnaire before end of March 2013. Two participants filled in the questionnaire end of May 2013, after being invited by other participants. (**D**) displays the influenza activity in the Netherlands in number of persons per 10000 inhabitants that visited the general practioner with influenza-like-illness (ILI) (as provided by NIVEL [47]).
(PDF)

Figure S2 **Graphical illustrations of contact definition and RDS recruitment.** (**A**) illustrates contact definition: a person sitting or standing within arm's length of the participant, which was denoted as “YourSpace”, for 30 seconds or longer. This figure was displayed in the online questionnaire to clarify to participants who they had to count as a contact person. (**B**) Figure illustrating difference between ‘contact persons’ and ‘recruitees’. We asked a participant (*P*) to invite four recruitees (*R*) who he/she had met according to the contact definition (within ‘YourSpace’) in the past 7 days. The blue circle illustrates ‘contact persons’ met by the participant 1 day before the day of filling in the questionnaire (‘yesterday’); these contact persons were recorded in the questionnaire. The green circle illustrates persons met 2–7 days before the day of filling in the questionnaire; we did not collect information on these persons. Participants could either have met recruitees ‘yesterday’ or 2–7 days before the participation day.
(PDF)

Figure S3 **Distributions of numbers of contact persons across days of the week by each location and all locations together.**
(PDF)

Figure S4 **Distributions of numbers of contact persons across days of the week by transport vehicle and all transport vehicles together.**
(PDF)

Figure S5 **Investigating the relation between the outcome and the independent (integer) variables.** The plots display the relation between the outcome and age, degree and household size. Plots **A** are based on the full Dutch sample (*n_Netherlands_* = 356); plots **B** are based on the Dutch sample without seeds (data without wave 0, *n_Netherlands_* = 264). Plots **C** are based on the full Thai sample (*n_Thailand_* = 251); plots **D** on the Thai sample without seeds (*n_Thailand_* = 163).
(PDF)

Table S1 **Subset of transport and location type of interest as shown in questionnaire.**
(PDF)

Table S2 **Symptoms displayed in questionnaire.**
(PDF)

Table S3 **Exploring the first-order Markov assumption: correlations in country samples.**
(PDF)

Text S1 **Drivers of online peer-recruitment: logistic regression.**
(PDF)

Text S2 **R source code.**
(R) Click here for additional data file.
