# Supplementary material for: Comparison of Contact Patterns Relevant for Transmission of Respiratory Pathogens in Thailand and the Netherlands Using Respondent-Driven Sampling
Source: PLoS One. 2014 Nov 25;9(11):e113711. doi: 10.1371/journal.pone.0113711 (PMC4244136; doi:10.1371/journal.pone.0113711)
Supplement: Figure S1 — Period of survey administration and influenza activity in each country. (A) displays the influenza activity in Thailand in numbers of specimens that were found positive for influenza (as provided by FluNet [46]). (B) Number of participants in Thailand collected between December 2012 and March 2013. (C) Number of participants in the Netherlands collected between end of November 2012 and beginning of May 2013. Most participants filled in the questionnaire before end of March 2013. Two participants filled in the questionnaire end of May 2013, after being invited by other participants. (D) displays the influenza activity in the Netherlands in number of persons per 10000 inhabitants that visited the general practioner with influenza-like-illness (ILI) (as provided by NIVEL [47]). (PDF) [file pone.0113711.s001.pdf]

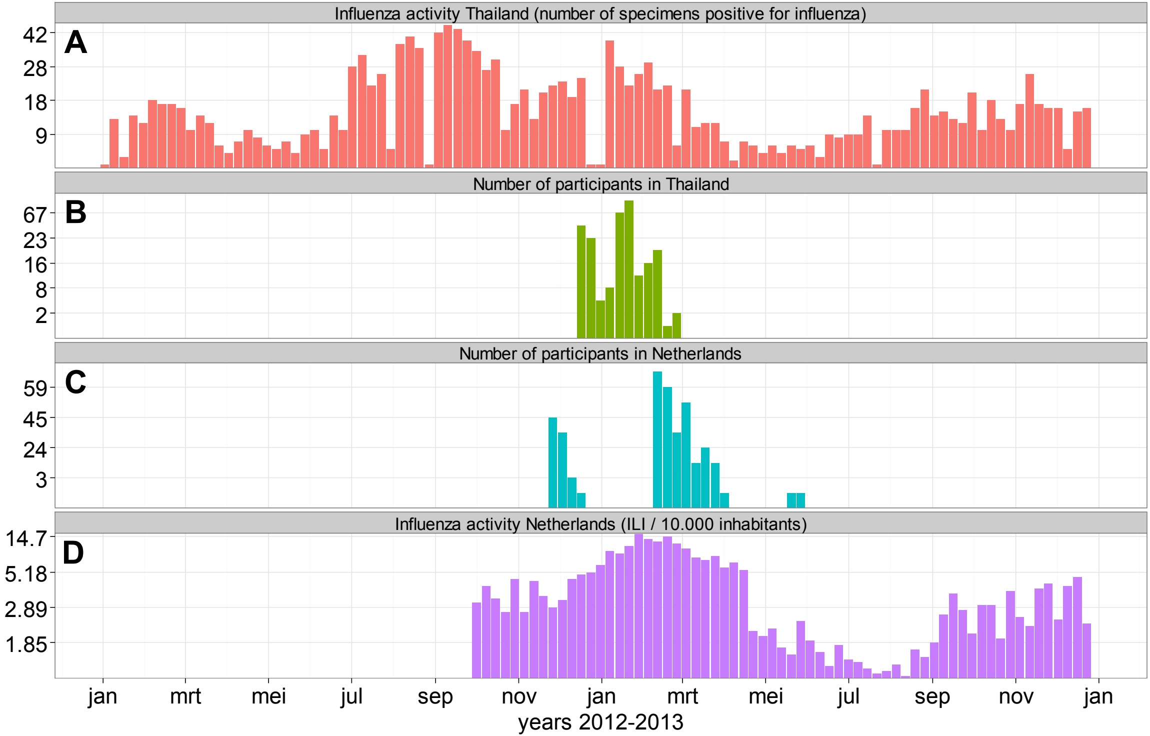

Influenza activity Thailand (number of specimens positive for influenza)
A
42
28
18
9
Number of participants in Thailand
B
67
23
16
8
2
Number of participants in Netherlands
C
59
45
24
3
Influenza activity Netherlands (ILI / 10.000 inhabitants)
D
14.7
5.18
2.89
1.85
jan
mrt
mei
jul
sep
nov
jan
mrt
mei
jul
sep
nov
jan
years 2012-2013
