# Supplementary figures and images for: Comparison of Contact Patterns Relevant for Transmission of Respiratory Pathogens in Thailand and the Netherlands Using Respondent-Driven Sampling
Source: PLoS One. 2014 Nov 25;9(11):e113711. doi: 10.1371/journal.pone.0113711 (PMC4244136; doi:10.1371/journal.pone.0113711)

**A**

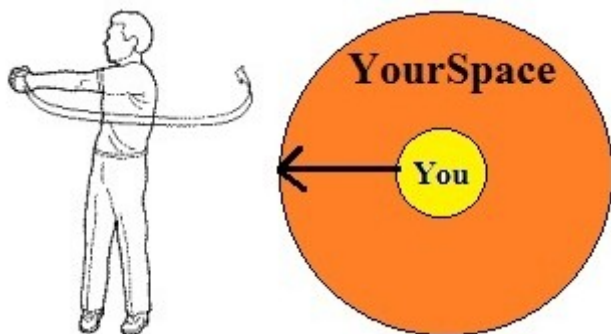

**B**

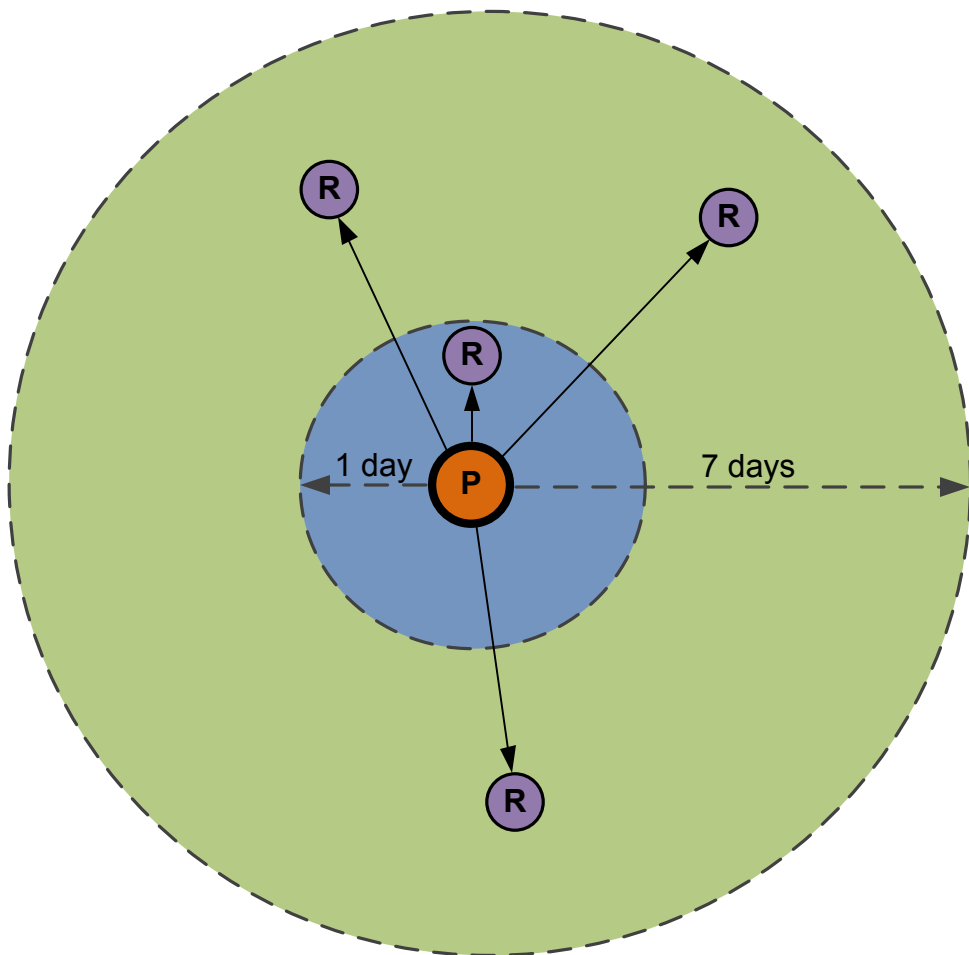

Supplement: Figure S2 — Graphical illustrations of contact definition and RDS recruitment. (A) illustrates contact definition: a person sitting or standing within arm's length of the participant, which was denoted as “YourSpace”, for 30 seconds or longer. This figure was displayed in the online questionnaire to clarify to participants who they had to count as a contact person. (B) Figure illustrating difference between ‘contact persons’ and ‘recruitees’. We asked a participant (P) to invite four recruitees (R) who he/she had met according to the contact definition (within ‘YourSpace’) in the past 7 days. The blue circle illustrates ‘contact persons’ met by the participant 1 day before the day of filling in the questionnaire (‘yesterday’); these contact persons were recorded in the questionnaire. The green circle illustrates persons met 2–7 days before the day of filling in the questionnaire; we did not collect information on these persons. Participants could either have met recruitees ‘yesterday’ or 2–7 days before the participation day. (PDF) [file pone.0113711.s002.pdf]

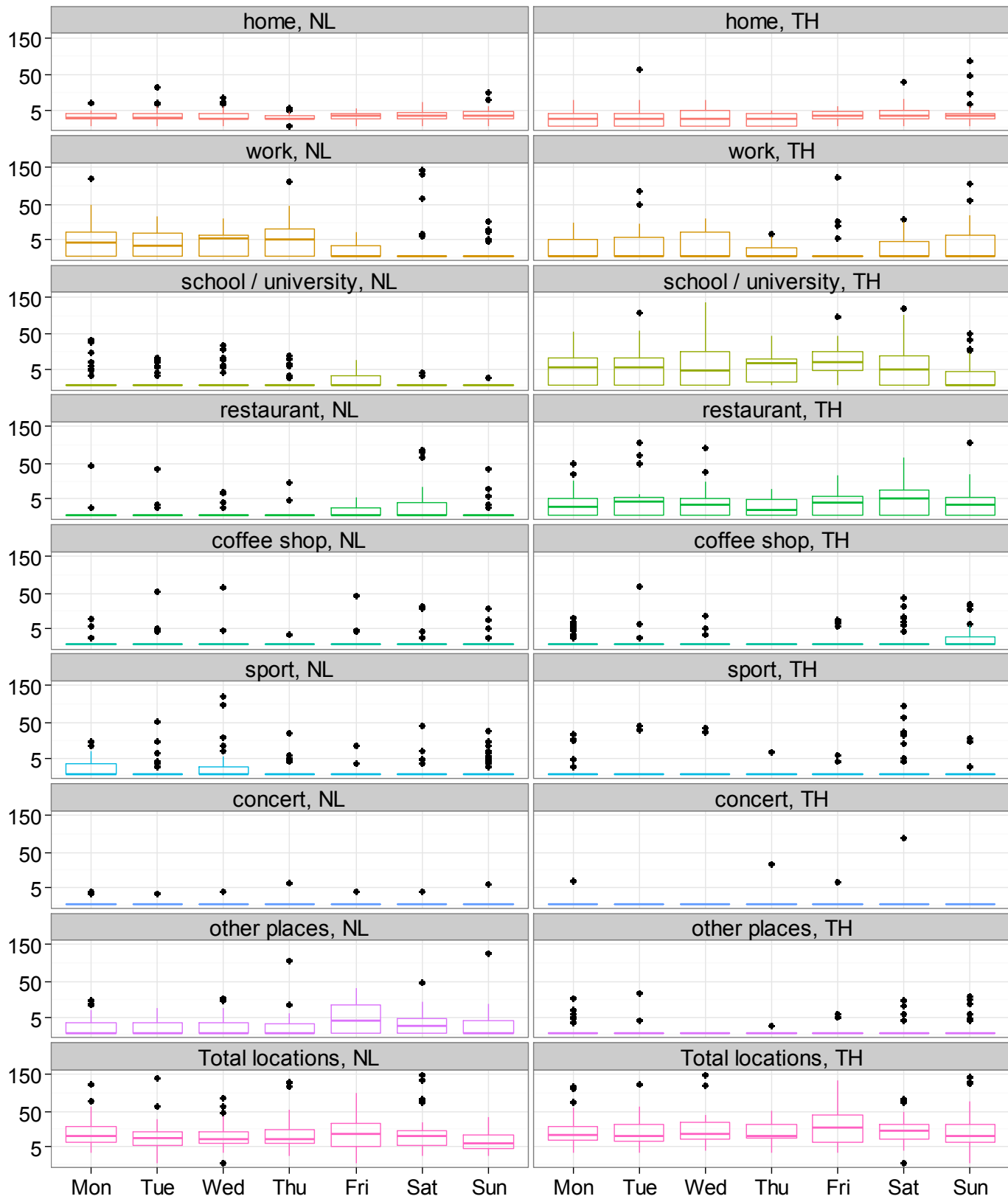

Supplement: Figure S3 — Distributions of numbers of contact persons across days of the week by each location and all locations together. (PDF) [file pone.0113711.s003.pdf]

Number of contact persons

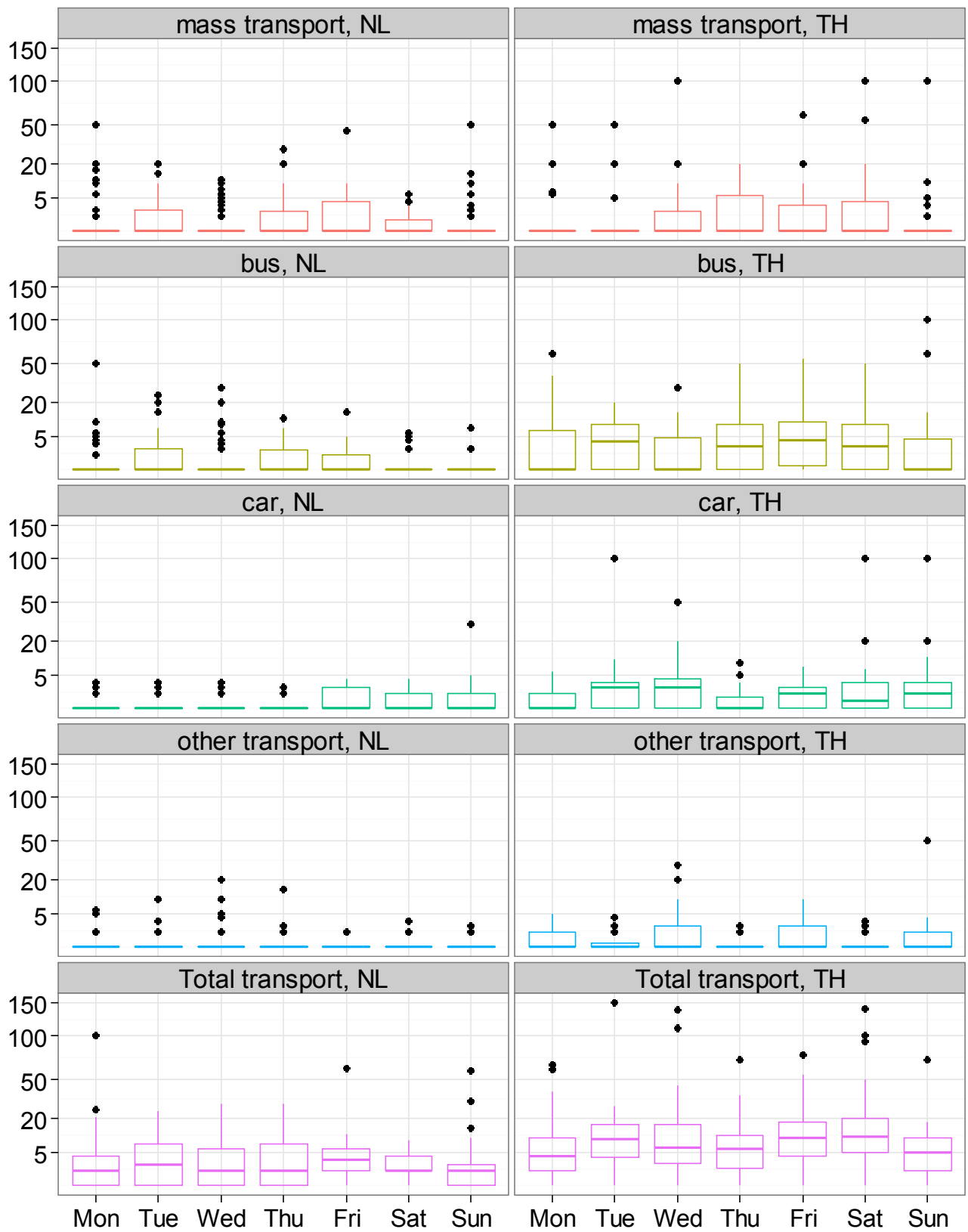

Supplement: Figure S4 — Distributions of numbers of contact persons across days of the week by transport vehicle and all transport vehicles together. (PDF) [file pone.0113711.s004.pdf]

**A**Netherlands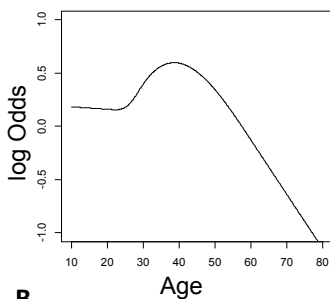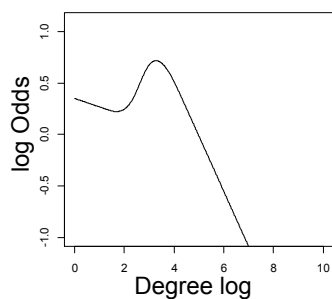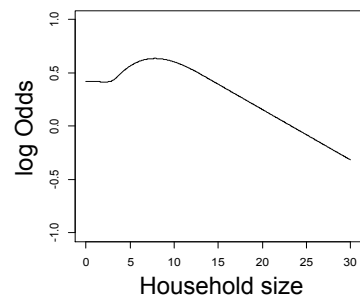**B**Netherlands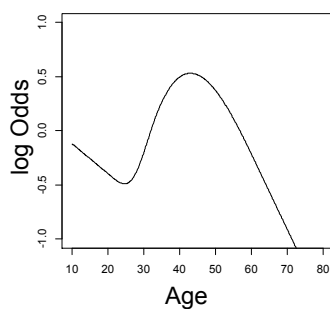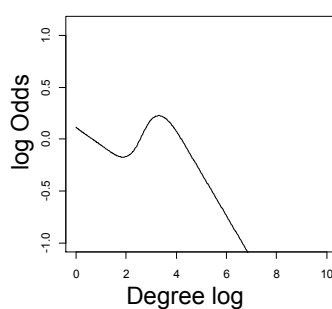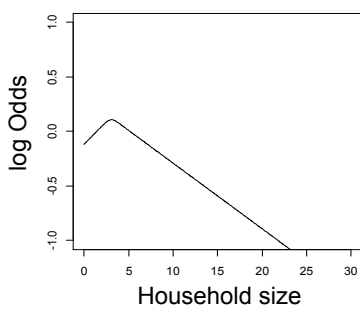**C**Thailand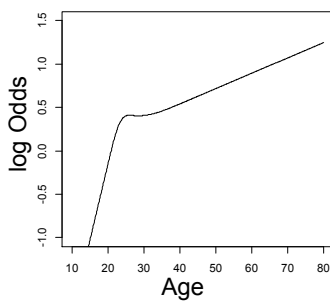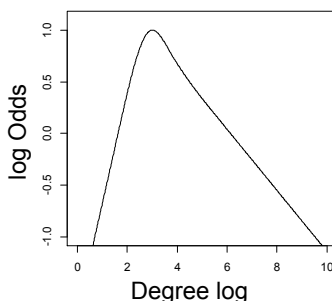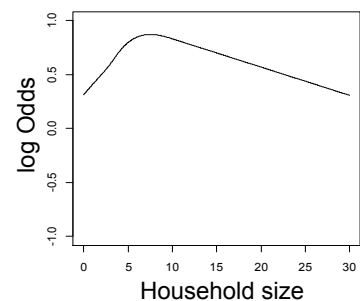**D**Thailand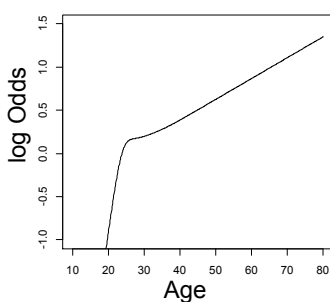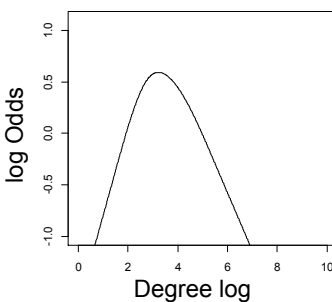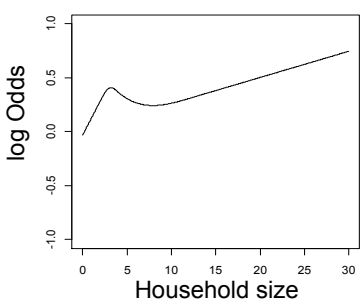

Supplement: Figure S5 — Investigating the relation between the outcome and the independent (integer) variables. The plots display the relation between the outcome and age, degree and household size. Plots A are based on the full Dutch sample (nNetherlands = 356); plots B are based on the Dutch sample without seeds (data without wave 0, nNetherlands = 264). Plots C are based on the full Thai sample (nThailand = 251); plots D on the Thai sample without seeds (nThailand = 163). (PDF) [file pone.0113711.s005.pdf]
