# Supplementary material for: Comparison of Contact Patterns Relevant for Transmission of Respiratory Pathogens in Thailand and the Netherlands Using Respondent-Driven Sampling
Source: PLoS One. 2014 Nov 25;9(11):e113711. doi: 10.1371/journal.pone.0113711 (PMC4244136; doi:10.1371/journal.pone.0113711)
Supplement: Table S1 — Subset of transport and location type of interest as shown in questionnaire. (PDF) [file pone.0113711.s006.pdf]

Table S1. Subset of transport and location type of interest as shown in questionnaire

	Displayed for participants in the Netherlands	Displayed for participants in Thailand
**Locations**	at home	at home
	at work	at work
	at school / university	at school / university
	in a restaurant	in a restaurant
	in a coffee shop	in a coffee shop
	at sports / leisure	at sports / leisure
	at a concert / theater / cinema	at a concert / theater / cinema
	at other places	at other places
**Transport vehicles**	Train and/or airplane	Skytrain and/or subway and/or airplane
	Bus and/or metro and/or tram	Bus and/or minibus and/or shuttle boot
	Car	Car and/or taxi
	Other transport vehicle	Motorbike and/or Tuk-Tuk
