# Supplementary material for: Comparison of Contact Patterns Relevant for Transmission of Respiratory Pathogens in Thailand and the Netherlands Using Respondent-Driven Sampling
Source: PLoS One. 2014 Nov 25;9(11):e113711. doi: 10.1371/journal.pone.0113711 (PMC4244136; doi:10.1371/journal.pone.0113711)
Supplement: Table S2 — Symptoms displayed in questionnaire. (PDF) [file pone.0113711.s007.pdf]

**Table S2. Symptoms displayed in questionnaire**

| Have you had any of the following complaints in the past week? |
|---|
| No complaints |
| Fever |
| Chills |
| Runny or blocked nose |
| Sore throat |
| Cough |
| Headache |
| Muscle/joint pain |
| Diarrhea |
| Other, namely |
