# Supplementary material for: Comparison of Contact Patterns Relevant for Transmission of Respiratory Pathogens in Thailand and the Netherlands Using Respondent-Driven Sampling
Source: PLoS One. 2014 Nov 25;9(11):e113711. doi: 10.1371/journal.pone.0113711 (PMC4244136; doi:10.1371/journal.pone.0113711)
Supplement: Table S3 — Exploring the first-order Markov assumption: correlations in country samples. (PDF) [file pone.0113711.s008.pdf]

Table S3. Exploring the first-order Markov assumption: correlations in country samples

	**Geodesic distance**[a]	**0 – 1**	**1 – 2**	**2 – 3**	**0 – 3**
**Netherlands**	**age** (*r*)	0.295 [0.131–0.443]*	0.614 [0.455–0.734]*	0.473 [0.160–0.699]£	0.171 [-0.177–0.481]
	**gender** (r_φ)	0.227 [0.059–0.383]£	0.382 [0.177–0.555]*	0.336 [-0.002–0.606]	0.106 [-0.241–0.428]
**Thailand**	**age** (*r*)	0.524 [0.350–0.663]*	0.621 [0.394–0.777]*	0.451 [0.011 – 0.745]£	0.320 [-0.143–0.668]
	**gender** (r_φ)	0.192 [-0.022–0.389]	0.347 [0.052–0.586]£	0.192 [-0.273 – 0.585]	0.000 [-0.443–0.443]

[a.] Link distance between seeds in wave 0 and contact persons in consecutive waves (waves 1 to 3).

* p-value < 0.001

£ p-value < 0.05
