# Supplementary material for: Comparison of Contact Patterns Relevant for Transmission of Respiratory Pathogens in Thailand and the Netherlands Using Respondent-Driven Sampling
Source: PLoS One. 2014 Nov 25;9(11):e113711. doi: 10.1371/journal.pone.0113711 (PMC4244136; doi:10.1371/journal.pone.0113711)
Supplement: Text S1 — Drivers of online peer-recruitment: logistic regression. (PDF) [file pone.0113711.s009.pdf]

Supplementary file of manuscript:
"Comparison of contact patterns relevant for transmission of respiratory pathogens in Thailand and the Netherlands using respondent-driven sampling"

Text S1. Drivers of online peer-recruitment: logistic regression.

We investigated which characteristics influence online peer-recruitment. We defined "intention to recruit" as a respondent that requested invitations for recruitees on the last survey page. To analyse which characteristics influence the intention to recruit (categorised as recruiter "requested" or "did not request" four invitations) we used logistic regression analyses, with intention to recruit as binary outcome. The log odds of the binary outcome was modeled as a linear combination of the variables degree (integer), age (integer), education (binary: higher or lower education), sex (binary) and household size (integer). We conducted this analysis for full samples obtained in the Netherlands and Thailand, as well as for the samples excluding seeds.

First, we investigated one by one whether the relations between the probability of requesting invitations (log odds = "LOG probability/1-probability") and the predictor variables (integer) were linear. We used "rcspline.plot" of the R package "Hmisc" to plot the estimated restricted cubic spline (RCS [1], with knots = 4) function, which relates a single variable - the "predictor"- to the outcome for a logistic model (see Figure S5). Knots were placed at fixed quantiles (0.05, 0.35, 0.65, 0.95) of the predictor's marginal distribution, ensuring that enough points are available in each interval and also guards against letting outliers overly influence knot placement [1]. We also used a Pearson's Chi-Squared test (with Yates' continuity correction) to analyse bivariate the independency between the outcome and the categorical predictors (Table I).

Figure S5 illustrates for both countries that the relations between the log odds of intention to recruit and the predictor variables age, degree (log transformed) and household were not linear. Log-transformation or other similar transformations did not result in linear relations. We therefore added age, degree (log) and household size with RCS (with knots = 3, as this resulted in a logistic model with the lowest Akaike's information criterion, AIC) to the logistic regression model. Table 1 shows for the Dutch sample that the outcome "intention to recruit" is dependent on the variable "sex". In order to compare both country samples and to analyse the relationship between degree and the intention to recruit (e.g. do individuals with a high degree have a higher probability to invite their recruitees compared to individuals with a low

degree?), we used a logistic regression model containing all “predictor” variables (i.e. a full model).

Table II shows the regression coefficients for the full Dutch sample. “rcs” indicate the cubic spline terms that we added to the model (*k*-1 regression parameters, not including the intercept). An RCS function with three knots includes two splines: S_0 (a linear part) and S_1 (a non-linear part). Estimates of $S_{>0}$ are virtually not interpretable [2].

In the Dutch sample, males had a significantly lower log odds (-0.629, p=0.009) of intending to invite recruitees compared to female participants when adjusted for degree, age, education and household size. We obtained similar results for the Dutch sample without seeds, see Table III. Although in the sample without seeds also age seemed to slighty influence the intention to recruit, besides sex. Table IV and V displays the regression coefficients for respectively the full Thai sample and the Thai sample without seeds. In the Thai sample, participants’ age and degree seemed to influence the intention to recruit.

We used the fitted logistic regression model to estimate probabilities of ‘intention to recruit’ *for individual subjects* as a function of degree. Thus, probabilities were estimated based on actual obtained data (‘data-driven’); not by keeping the other predictors ‘constant’ by using means. Confidence intervals (95%) were obtained using standard errors. Lines and confidence intervals in Figure 3d were smoothed using ‘geom_smooth’ of the R-package “ggplot2”.

Literature

1. Harrell F.E. 2001 Restricted Cubic Splines. In *Regression Modeling Strategies: With Applications to Linear Models, Logistic Regression, and Survival Analysis* (ed. Springer), pp. 20-26. New York, Springer-Verlag New York.
2. Desquilbet L., Mariotti F. 2010 Dose-response analyses using restricted cubic spline functions in public health research. *Statistics in medicine* **29**(9), 1037-1057. (doi:10.1002/sim.3841).

Table I. Chi-squared test to analyse independence by outcome variable.

		Chi-squared	df	p-value
The Netherlands	Sex	5.4153	1	0.01996
	Education	0	1	1
Thailand	Sex	0.0053	1	0.9421
	Education	0.0032	1	0.9549

Table II. Output of logistic regression for the Netherlands, full sample

	estimate[a]	SE	z value	Pr(>|z|)	2.50%	97.50%
Constant	-1.009	1.292	-0.781	0.435	-3.542	1.524
rcs degree(log)_S0	0.230	0.221	1.039	0.299	-0.204	0.664
rcs degree(log)_S1	-0.274	0.271	-1.013	0.311	-0.805	0.256
rcs age_S0	0.054	0.043	1.242	0.214	-0.031	0.138
rcs age_S1	-0.149	0.114	-1.300	0.194	-0.373	0.076
higher education	-0.216	0.415	-0.521	0.603	-1.030	0.598
male	-0.629	0.241	-2.613	0.009	-1.100	-0.157
rcs household size_S0	0.024	0.193	0.123	0.902	-0.355	0.403
rcs household size_S1	-0.028	0.291	-0.097	0.923	-0.598	0.542

[a]Null deviance: 434.91 (df: 324); residual variance: 425.18 (df: 316) and AIC 443.18.

Table III. Output of logistic regression for the Netherlands, sample without seeds

	estimate[a]	SE	z value	Pr(>|z|)	2.50%	97.50%
Constant	-2.255	1.398	-1.613	0.107	-4.994	0.485
rcs degree(log)_S0	0.155	0.270	0.574	0.566	-0.374	0.685
rcs degree(log)_S1	-0.232	0.366	-0.634	0.526	-0.949	0.485
rcs age_S0	0.089	0.046	1.912	0.056	-0.002	0.180
rcs age_S1	-0.216	0.131	-1.651	0.099	-0.473	0.040
higher education	-0.443	0.441	-1.004	0.315	-1.307	0.421
male	-0.625	0.279	-2.240	0.025	-1.171	-0.078
rcs household size_S0	0.089	0.219	0.408	0.683	-0.340	0.519
rcs household size_S1	-0.155	0.336	-0.461	0.645	-0.814	0.504

[a]Null deviance: 327.17 (df: 235); residual variance: 316.36 (df: 227) and AIC 334.36.

Table IV. Output of logistic regression for Thailand, full sample

	estimate[a]	SE	z value	Pr(>|z|)	2.50%	97.50%
Constant	-4.122	1.906	-2.162	0.031	-7.859	-0.385
rcs degree(log)_S0	0.645	0.281	2.299	0.022	0.095	1.195
rcs degree(log)_S1	-1.189	0.441	-2.697	0.007	-2.053	-0.325
rcs age_S0	0.135	0.075	1.804	0.071	-0.012	0.281
rcs age_S1	-0.121	0.093	-1.301	0.193	-0.303	0.061
higher education	-0.738	0.513	-1.439	0.150	-1.744	0.267
male	0.150	0.318	0.471	0.637	-0.474	0.774
rcs household size_S0	0.131	0.157	0.833	0.405	-0.177	0.439
rcs household size_S1	-0.008	0.153	-0.051	0.960	-0.307	0.292

[a]Null deviance: 278.50 (df: 216); residual variance: 262.11 (df: 208) and AIC 280.11

Table V. Output of logistic regression for Thailand, sample without seeds

	estimate[a]	SE	z value	Pr(>|z|)	0.025	0.975
Constant	-6.236	2.511	-2.483	0.013	-11.157	-1.314
rcs degree(log)_S0	0.566	0.335	1.688	0.091	-0.091	1.224
rcs degree(log)_S1	-0.974	0.541	-1.801	0.072	-2.035	0.086
rcs age_S0	0.231	0.107	2.155	0.031	0.021	0.440
rcs age_S1	-0.238	0.136	-1.742	0.081	-0.505	0.030
higher education	-0.898	0.710	-1.264	0.206	-2.289	0.494
male	0.089	0.391	0.228	0.819	-0.678	0.856
rcs household size_S0	0.036	0.180	0.199	0.842	-0.318	0.390
rcs household size_S1	0.061	0.168	0.364	0.716	-0.268	0.390

[a]Null deviance: 186.69 (df: 136); residual variance: 172.35 (df: 128) and AIC 190.35.
